# Nucleotide-Binding Oligomerization Domain-1 and -2 Play No Role in Controlling *Brucella abortus* Infection in Mice

**DOI:** 10.1155/2012/861426

**Published:** 2011-11-30

**Authors:** Fernanda S. Oliveira, Natalia B. Carvalho, Dario S. Zamboni, Sergio C. Oliveira

**Affiliations:** ^1^Laboratory of Immunology of Infectious Diseases, Department of Biochemistry and Immunology, Institute of Biological Sciences, Federal University of Minas Gerais, 31270-901 Belo Horizonte, MG, Brazil; ^2^Department of Cell Biology, School of Medicine of Ribeirão Preto-SP, University of São Paulo (FMRP/USP), 14049-900 Ribeirão Preto, SP, Brazil

## Abstract

Nucleotide-binding oligomerization domain proteins (NODs) are modular cytoplasmic proteins implicated in the recognition of peptidoglycan-derived molecules. Further, several *in vivo* studies have demonstrated a role for Nod1 and Nod2 in host defense against bacterial pathogens. Here, we demonstrated that macrophages from NOD1-, NOD2-, and Rip2-deficient mice produced lower levels of TNF-*α* following infection with live *Brucella abortus* compared to wild-type mice. Similar reduction on cytokine synthesis was not observed for IL-12 and IL-6. However, NOD1, NOD2, and Rip2 knockout mice were no more susceptible to infection with virulent *B. abortus* than wild-type mice. Additionally, spleen cells from NOD1-, NOD2-, and Rip2-deficient mice showed unaltered production of IFN-*γ* compared to C57BL/6 mice. Taken together, this study demonstrates that NOD1, NOD2 and Rip2 are dispensable for the control of *B. abortus* during *in vivo* infection.

## 1. Introduction

The innate immune system plays a crucial role in host defense against invading pathogens and relies on pattern recognition receptors (PRRs), which detect conserved microbial- or danger-associated molecular patterns (MAMPs or DAMPs). Several PRRs have been identified, among them are the TLRs (Toll-like receptors), NLRs (nucleotide-binding and oligomerization domain-like receptors), and RLR (retinoic-acid-inducible gene-1-like receptors) [1].

Nod1 and Nod2 are NLR proteins that trigger nuclear factor-*κ*B (NF-*κ*B) signaling in response to bacterial peptidoglycan. Specifically, Nod1 recognizes muramyl peptides containing *meso*-DAP (diaminopimelic acid) found in the peptidoglycan of most Gram-negative bacteria and certain Gram-positive bacteria [2] whereas Nod2 recognizes muramyl dipeptide (MDP) produced in all bacteria [3]. Upon peptidoglycan detection, Nod1 and Nod2 recruit and associate with the adaptor protein Rip2, triggering proinflammatory pathways such as NF-*κ*B and the mitogen-activated protein (MAP) kinases p38, JNK, and ERK [4]. Furthermore, activation of Nod1 and Nod2 by live bacteria triggers proinflammatory responses, leading to the induction of cytokine and chemokines [5, 6]. Using Nod-deficient mice, several *in vivo* studies have demonstrated a role for Nod1 and Nod2 in host defense against pathogens such as *Helicobacter pylori, Listeria monocytogenes, Staphylococcus aureus, and Legionella pneumophila* [7–10].

*Brucella* is a Gram-negative bacterium which is pathogenic of human and animals [11]. The immune response against *Brucella* infection involves many molecules and cells to trigger a Th1 immune response and activation of CD8+ *T* cells [12–14]. The innate immune response against *B. abortus* infection begins with the recognition of molecular structures related to this pathogen by receptors such as Toll-like receptors (TLRs) [15]. Some *in vitro* and *in vivo* studies have shown the involvement of TLR2, TLR4, and TLR9 in the recognition of *Brucella* and induction of inflammatory response [16–20]. Moreover, our group and others have demonstrated that MyD88 is essential for host control of *Brucella* infection *in vivo* and the induction of proinflammatory cytokines [21]. So far, no study has demonstrated the role of NOD-like receptors in the control of *Brucella* infection. Herein, we have shown that NOD1, NOD2, or the adaptor molecule Rip2 plays no role in enhancing resistance to *B. abortus* infection *in vivo*. However, reduced production of TNF-*α* was detected in bone-marrow-derived macrophages (BMDM) from NOD1, NOD2, and Rip2 KO mice compared to C57BL/6.

## 2. Materials and Methods

### 2.1. Mice

NOD1, NOD2, and RIP2 genetically deficient mice (NOD1^−/−^, NOD2^−/−^, and RIP2^−/−^) were kindly gifted by Dr. Richard Flavell (Yale University) and maintained in the animal facility of the University of São Paulo (FMRP/USP). The wild-type strain C57BL/6 mice were purchased from the Federal University of Minas Gerais (UFMG, Belo Horizonte, Brazil). Wild-type and deficient mice were maintained at UFMG and used at 6–8 weeks of age.

### 2.2. Bacteria

*Brucella abortus* virulent strain S2308 was obtained from our laboratory collection [22]. The strain S2308 was grown in *Brucella* Broth liquid medium (BB) (DIFCO) at 37°C under constant agitation. After three days of growth, the bacterial culture was centrifuged and the pellet was resuspended in phosphate buffered saline (PBS) 0.15 M pH 7.4 (2.8 Na_2_PO_4_ mM, 7.2 mM Na_2_HPO_4_, and 0.14 M NaCl). Aliquots of these cultures were serially diluted and plated on BB medium containing 1.5% bacteriological agar. After incubation for 72 hours at 37°C, bacterial numbers were determined by counting colony forming units (CFU).

### 2.3. *B. abortus* Infection

Five mice from each group C57BL/6, NOD1^−/−^, NOD2^−/−^, and RIP2^−/−^ were infected intraperitoneally with 1 × 10^6^ CFU of *B. abortus* strain S2308. These mice were sacrificed at 2 weeks after infection. The spleen harvested from each animal was macerated in 10 mL of saline (NaCl 0.8%), and it was used for counting of CFU and splenocyte culture. To count residual *Brucella* CFU, spleen cells were serially diluted and were plated in duplicate on BB agar. After 3 days of incubation at 37°C, the number of colony forming units (CFU) was determined. Results were expressed as the mean log CFU of each group.

### 2.4. Measurement of IFN-*γ* into Splenocyte Culture Supernatants

Spleens cells from C57BL/6, NOD1^−/−^, NOD2^−/−^, and RIP2^−/−^ mice were treated with ACK buffer (0.15 M NH_4_Cl, 1.0 mM KHCO_3_, 0.1 mM Na_2_EDTA, pH 7.2) to lyse red blood cells. After that, the cells were washed with saline (NaCl 0.8%) and suspended in RPMI 1640 (Gibco, Carlsbad, Calif, USA) supplemented with 2 mM L-glutamine, 25 mM HEPES, 10% heat-inactivated FBS (Gibco, Carlsbad, CA), penicillin G sodium (100 U/mL), and streptomycin sulfate (100 *μ*g/mL). To determine cytokine concentration by ELISA, 1 × 10^6^ spleen cells were plated per well in a 96-well tissue culture-treated dish. Splenocytes were stimulated with *B. abortus* S2308 (MOI 100 : 1) or concanavalin A (5 *μ*g/mL Sigma, Sigma-Aldrich, St. Louis, Mo, USA). Unstimulated cells were used as negative control. Spleen cells were incubated at 37°C in 5% CO_2_ for 72 h, after that supernatants were harvested for measuring IFN-*γ* levels. IFN-*γ* was measured into cell supernatants by ELISA using the Duoset kit (R&D Systems, Minneapolis, Minn, USA) according to the manufacturer's instructions. 

### 2.5. Generation and In Vitro Stimulation of Bone-Marrow-Derived Macrophages- (BMDMs)

Macrophages were derived from bone marrow of C57BL/6, NOD1^−/−^, NOD2^−/−^, and RIP2^−/−^mice as previously described [23]. Briefly, bone marrow (BM) cells were removed from the femurs and tibias of the animals and cultured in DMEM (Gibco, Carlsbad, Calif, USA) containing 10% FBS (HyClone, Logan, Utah, USA), 1% HEPES, and 10% L929 cell-conditioned medium (LCCM) as source of M-CSF, in 24-well plates (5 × 10^5^ cells/well). After 4 days, 100 *μ*L/well LCCM was added. At day 7, the medium was renewed. At day 10 of culture, when the cells had completely differentiated into macrophages, the medium was harvested and we added supplemented DMEM (500 *μ*L/well) containing *B. abortus* S2308 (MOI 1000 : 1) or *E. coli *LPS (1 *μ*g/mL, Sigma, St. Louis, Mo, USA). Culture supernatants of BMDMs were collected after 24 hours of stimulation and assayed for the concentrations of IL-12, IL-6, and TNF-*α* by ELISA (R&D Systems) according to the manufacturer's instructions.

### 2.6. Statistical Analysis

A previous analysis of normal distribution of the data was performed, and ANOVA was used followed by Tukey's test when we compared more than two variables. Furthermore, Student's *t-*test was applied when only two variables were compared using GraphPad Prism 4 (GraphPad Software, Inc.). The level of significance in the analysis was *P* < 0.01.

## 3. Results

### 3.1. NOD1, NOD2, and Rip2 KO Mice Control *B. abortus* Infection

**Figure 1:**
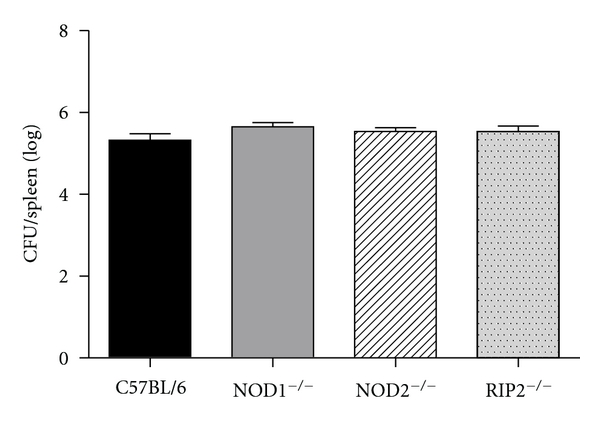
Control of *Brucella abortus* infection is NOD1 and NOD2 independent. C57BL/6, NOD1^−/−^, NOD2^−/−^, and RIP2^−/−^ mice were intravenously infected with 10^6^ CFU of *B. abortus* S2308, and the number of bacteria in the spleen was analyzed by counting CFU at 2 weeks after infection. Data are expressed as mean ± SD of five animals per time point.

To investigate the role of NOD1, NOD2, and Rip2 molecules during *B. abortus *infection, knockout and wild-type mice were infected with 1 × 10^6^ CFU of *B. abortus* strain S2308 and the number of bacteria in mouse spleens was monitored by colony forming units (CFU) counting. As shown in Figure 1, there was no difference in bacterial load from NOD1, NOD2, and Rip2 KO mice compared to C57BL/6. These results indicate that NOD1, NOD2, and Rip2 are not important to *in vivo* host control of *Brucella*.

### 3.2. NOD1, NOD2, and Rip2 Do Not Account for IFN-*γ* Response to *B. abortus*

**Figure 2:**
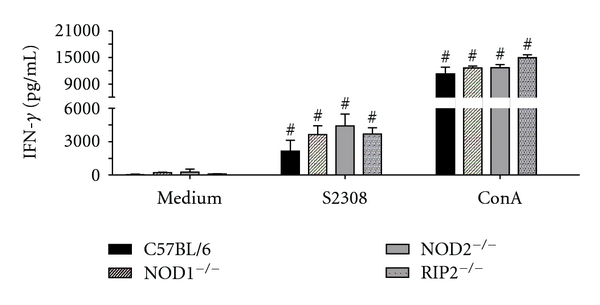
IFN-*γ* production by spleen cells induced by *B. abortus* in NOD1, NOD2, and Rip2 KO mice. C57BL/6, NOD1^−/−^, NOD2^−/−^, and RIP2^−/−^ mice were infected with 10^6^ CFU of *B. abortus* S2308, and 2 weeks after infection, spleen cells (1 × 10^6^/well) were stimulated with *B. abortus* S2308 (MOI 100 : 1) or concanavalin A (5 *μ*g/mL). Supernatants were harvested after 72 h for measuring IFN-*γ* levels by ELISA. Statistically significant difference in relation to nonstimulated cells is denoted with #. The significance of differences was compared by ANOVA followed by Tukey's test (*P* < 0.01).

Protective immunity against infection by *B. abortus* is directly related to the induction of a type 1 pattern of immune response [24]. IFN-*γ* is a critical cytokine involved in this type of immunity. Thus, to evaluate the role of NOD1, NOD2, and Rip2 in inducing a type 1 immune response during *B. abortus* infection, splenocytes from *Brucella*-infected animals were stimulated with live *B. abortus*. After 72 hrs of cell culture, the supernatant was collected and the level of IFN-*γ* was analyzed. Herein, it was observed a similar level of IFN-*γ* production by NOD1, NOD2, or Rip2 KO mice when compared to wild-type animals (Figure 2). Taken together, these results suggest that the lack of NOD1, NOD2, and Rip2 causes no effect on induction of type 1 immune response by *B. abortus*. 

### 3.3. Lack of NOD1, NOD2, and Rip2 Causes a Significant Reduction in TNF-*α* Production by Macrophages

**Figure 3:**
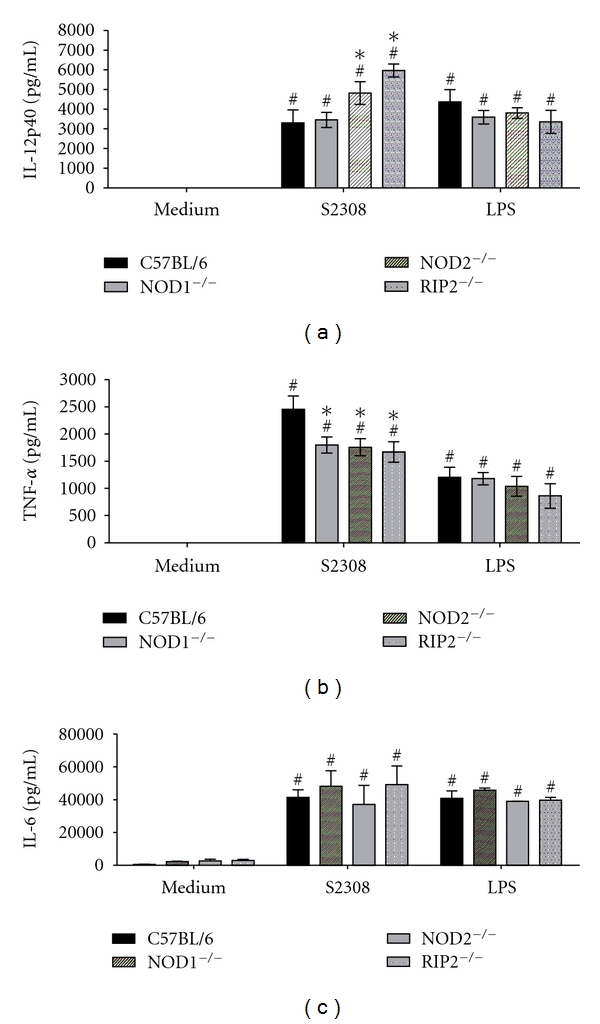
TNF-*α* production induced by *B. abortus* in macrophages, but not IL-12 and IL-6, requires NOD1, NOD2, and Rip2. Bone marrow from C57BL/6, NOD1^−/−^, NOD2^−/−^, and RIP2^−/−^ mice cells was differentiated in macrophages and stimulated with *B. abortus* S2308 (MOI 100 : 1) or *E. coli* LPS (1 *μ*g/mL). Supernatants was harvested for measuring IL-12 (a), TNF-*α* (b), and IL-6 (c) after 24 hrs by ELISA. Statistically significant difference in relation to non-stimulated cells is denoted with # and in relation to C57BL/6 mice is denoted with an asterisk (∗). The significance of differences was compared by ANOVA followed by Tukey's test (*P* < 0.01).

The recognition of *Brucella* by innate immunity cells, such as macrophages and dendritic cells, results in activation and the concomitant production of proinflammatory cytokines [19]. In order to evaluate the role of NOD1, NOD2, or Rip2 in the proinflammatory cytokine production, bone-marrow-derived macrophages from NOD1, NOD2, or Rip2 KO and C57BL/6 mice were stimulated with *B. abortus*. As shown in Figure 3(b), NOD1, NOD2, or Rip2 deficiency reduced the production of TNF-*α* by macrophages from knockout mice compared to wild type cells. In contrast, IL-12 levels of NOD1 KO cells remained unaltered compared to wild type but were higher in supernatants of NOD2 and Rip2 KO macrophages (Figure 3(a)). Regarding IL-6, the levels of this cytokine produced by knockout macrophages were similar to C57BL/6 cells (Figure 3(c)). These results showed that NOD1, NOD2, and Rip2 are important molecules involved in TNF-*α* synthesis induced by *Brucella* in macrophages but not in IL-6 and IL-12 production.

## 4. Discussion

Innate immune responses against intracellular pathogens are crucial to produce an efficient host response that triggers control of microbial replication and resistance to infection. NOD receptors are important molecules that play a key role in the induction of nitric oxide, a molecule that is known to be directly microbicidal [25]. Further, activation of NOD1 and NOD2, by live bacteria triggers proinflammatory responses, leading to the induction of cytokine and chemokine [5, 6]. In this study, we aimed to analyze the contribution of NOD1, NOD2, and Rip2 to the immune responses triggered by the intracellular bacterium *B. abortus*. 

To examine the role of NOD1, NOD2, and Rip2 in control of *B. abortus in vivo*, we used knockout mice for these molecules. At 2-weeks after-infection, no significant differences in *B. abortus* CFU were observed between C57BL/6 and NOD1^−/−^, NOD2^−/−^, and Rip2^−/−^ (Figure 1). We next examined the participation of NOD1, NOD2, and Rip2 in IFN-*γ* production during *B. abortus* infection. As observed in Figure 2, the level of IFN-*γ* produced by NOD1^−/−^, NOD2^−/−^, and Rip2^−/−^ spleen cells was not different from wild-type mice. Consistent with these results, Rip2 was found to be dispensable for the induction of an effective Th1 response during *Toxoplasma gondii* infection [26], and mice double knockout to Nod1 and Nod2 respond similarly to wild type to restrict protozoan parasite infection by *Plasmodium berghei* [27]. Similarly, single deficiency in NOD1 or NOD2 had little or no effect on restriction of bacterial growth inside host cells during *L. pneumophila* or *M. tuberculosis* infection [10, 28]. In the case of *L. pneumophila*, NLR-dependent bacterial recognition triggers early responses that are further sustained by TLRs signaling pathways [29]. *Brucella* possesses both TLR and NLR agonists; however, it seems that they do not act synergistically to activate host cells. In situations where cells are rendered refractory to TLR agonists, it is possible that NOD1/2 signaling is increased [30]. Here, we speculate that during host responses to some pathogens that are strongly TLR dependent, NLRs become minor components of the pathogen recognition machinery. According to this hypothesis, we have previously determined the critical role of TLR/MyD88 axis to host control of *Brucella* infection [19].

Macrophages are key elements in the innate immune response and recognition of *Brucella* components resulting in the production of proinflammatory cytokines [19]. Herein, we investigated the involvement of NOD1, NOD2, and Rip2 in *Brucella*-induced IL-12, IL-6, and TNF-*α* by macrophages. Macrophages deficient in NOD1, NOD2, and Rip2 showed reduced production of TNF-*α*, but not IL-6, when they were stimulated with live *Brucella* as compared to C57BL/6 cells. Unexpectedly, we detected enhanced production of IL-12 for NOD2 and Rip2 KO macrophages. Berrington et al. [31] have also observed increased IL-6 and MCP-1 levels in NOD1 and NOD2 KO lung cells infected with *L. pneumophila*. They suggested that NOD1 and NOD2 regulate proinflammatory cytokine response by an unknown mechanism. One possibility is that, through heterotypic association of the caspase-1 recruitment domains, NOD1/NOD2 may inhibit inflammasome components or modulate cytokine production through interaction with TLR-pathway intermediates [32].

Taken together, the findings of this study provide evidence that NOD1, NOD2, and Rip2 may participate in innate immune signaling in response to *B. abortus*, but they are not essential for host defense against *B. abortus* infection *in vivo*. To the best of our knowledge, this is the first report that demonstrates the dispensable role of NOD1 and NOD2 to control *Brucella* infection.
